# Adenylate kinase potentiates the capsular polysaccharide by modulating Cps2D in *Streptococcus pneumoniae* D39

**DOI:** 10.1038/s12276-018-0141-y

**Published:** 2018-09-05

**Authors:** Prachetash Ghosh, Truc Thanh Luong, Masaud Shah, Trung Thanh Thach, Sangdun Choi, Sangho Lee, Dong-Kwon Rhee

**Affiliations:** 1 School of Pharmacy, Sungkyunkwan University, Suwon, 16419 Korea; 2 Department of Molecular Science and Technology, Ajou University, Suwon, Gyeonggi-do 16499 Korea; 3 Department of Biological Sciences, Sungkyunkwan University, Suwon, 16419 Korea

## Abstract

*Streptococcus pneumoniae* is a polysaccharide-encapsulated bacterium. The capsule thickens during blood invasion compared with the thinner capsules observed in asymptomatic nasopharyngeal colonization. However, the underlying mechanism regulating differential CPS expression remains unclear. CPS synthesis requires energy that is supplied by ATP. Previously, we demonstrated a correlation between ATP levels and adenylate kinase in *S. pneumoniae* (SpAdK). A dose-dependent induction of SpAdK in serum was also reported. To meet medical needs, this study aimed to elucidate the role of SpAdK in the regulation of CPS production. CPS levels in *S. pneumoniae* type 2 (D39) increased proportionally with SpAdK levels, but they were not related to pneumococcal autolysis. Moreover, increased SpAdK levels resulted in increased total tyrosine kinase Cps2D levels and phosphorylated Cps2D, which is a regulator of CPS synthesis in the D39 strain. Our results also indicated that the SpAdK and Cps2D proteins interact in the presence of Mg-ATP. In addition, in silico analysis uncovered the mechanism behind this protein–protein interaction, suggesting that SpAdK binds with the Cps2D dimer. This established the importance of the ATP-binding domain of Cps2D. Taken together, the biophysical interaction between SpAdK and Cps2D plays an important role in enhancing Cps2D phosphorylation, which results in increased CPS synthesis.

## Pneumococcal disease: enzyme interaction promotes thickening of defensive bacterial capsule

A physical interaction between two key enzymes explains how the bacterium responsible for causing pneumococcal disease thickens its external capsule during infection of the bloodstream. A team led by Dong-Kwon Rhee from Sungkyunkwan University in Suwon, South Korea, studied strains of *Streptococcus pneumoniae* expressing varying levels of an enzyme that helps maintain the proper balance of cellular energy. They found that this enzyme stimulated the production of sugar chains that coat the outside of the bacterial capsule by binding and activating an intermediary enzyme involved in the synthesis of these sugar chains. Since the capsule is critical in warding off the human immune response, the findings suggest that drugs designed to disrupt the enzyme-mediated induction of capsule formation could help prevent or treat pneumococcal disease.

## Introduction

*Streptococcus pneumoniae* (a pneumococcus) colonizes the human respiratory tract as a commensal. It is also responsible for local infections and a number of invasive diseases such as pneumonia, sepsis, and meningitis^1–3^. More than 90 serotypes of pneumococcus have been reported. Migration through tissue barriers and subsequent adaptation to diverse host niches have converted an apparently harmless commensal into an invasive pathogen. This adaptation process is multifactorial and occurs in an extremely regulated manner^4^.

Some pneumococci are encapsulated by capsular polysaccharides (CPSs), which are important virulence factors that protect bacteria from the host immune system^5^. The ability of *S. pneumoniae* to control CPS production plays an important role in pneumococcal survival in various host niches^6^. However, the mechanism underlying the regulation of differential CPS production is not properly understood^7^. Previous studies showed that, with the exception of type 37, pneumococcal CPS production mostly relies on the *cps* locus positioned between the *dexB* and *aliA* genes^8,9^. In the pneumococcal *cps* locus, the first four genes are highly conserved and are designated *cpsA* (transcription activator), *cpsB* (phosphotyrosine-protein phosphatase), *cpsC* (polysaccharide export protein), and *cpsD* (autophosphorylating tyrosine kinase)^10–12^. The genes downstream of *cpsD* are serotype specific^7^ and are mainly responsible for polymerization and dissemination of polysaccharide^13^. In the type 2 pneumococcal *cps* locus, these genes are denoted as *cps2A*, *cps2B*, *cps2C*, and *cps2D*^14^. Cps2B, Cps2C, and Cps2D constitute a phosphoregulatory system that partly modulates CPS levels^10–12^.

In all organisms including pneumococci, intracellular ATP regulation is considered one of the most important factors for survival^15,16^. *S. pneumoniae* adenylate kinase (SpAdK) was crystalized and found to be essential for pneumococcal growth^3^. SpAdK catalyzes the conversion between adenylate nucleotides: Mg · ATP + AMP ↔ Mg · ADP + ADP^17^. Furthermore, SpAdK is essential for pneumococcal viability^3^. BLAST searches indicated a highly conserved *adk* sequence among various pneumococcal serotypes^3^, suggesting that the *adk* gene may be a potent target for developing serotype-independent chemotherapeutics.

Although antimicrobial therapies and conjugate vaccines are available, *S. pneumoniae* continues to be the etiologic agent of various diseases. Intensive efforts are focused on encouraging the development of alternative pneumococcal vaccine approaches that overcome the drawbacks of the present strategies while maintaining efficacy. CPS is one of the most significant pneumococcal virulence factors both in the context of disease pathogenesis and therapeutic importance. This is because all commercially available pneumococcal vaccines have been developed on the basis of capsular serotypes^18^. The structural and chemical variability of capsules among different pneumococcal strains is responsible for the fundamental limitations of CPS-based vaccines, including serotype-specific protection, capsular switching, and serotype replacement^19–21^. The capsule is an energetically expensive structure to produce, because the carbohydrates that are assimilated could otherwise be used in glycolysis to facilitate replication^22^. Therefore, it is expected that the capsule biosynthesis machinery is linked with pneumococcal metabolic regulatory systems^23^.

Previously we showed that the fucose-inducible conditional encapsulated (S type) *adk* mutant was not able to grow, as well as the isogenic non-encapsulated (R type) *adk* mutant, possibly due to the excess ATP required for CPS production. SpAdK expression increased in a serum dose-dependent manner^3^. Although the reduced virulence of the *adk* mutant compared with the wild-type (WT) was also observed in mice^3^, the underlying mechanism remains unknown. On the other hand, maximum expression of CPS was observed during systemic virulence^6^. Expression of *cps2A* was also induced in the presence of serum^24^. Thus, we studied the role of SpAdK in pneumococcal CPS synthesis.

Our results indicated that *S. pneumoniae* CPS increased proportionally with SpAdK levels. In addition, the increase in SpAdK expression was correlated with phosphorylation of Cps2D, a regulator of CPS synthesis. Moreover, SpAdK interacted physically with Cps2D in the presence of Mg-ATP. The autophosphorylation mechanism of CpS2D and interaction of SpAdK with Cps2D were further supported using in silico analyses.

## Materials and methods

### Ethical statement and raising antibodies

This is described in the Supplementary Information

### Bacterial strains, culture conditions, cloning, and protein purification

All reagents used for bacterial culture were purchased from Difco BD (NJ, USA). The bacterial strains and plasmids used in this study are listed in Table 1. Non-encapsulated *S. pneumoniae* serotype 2 CP1200^25^, R6 (a derivative of D39), and TTL04 were cultured in casitone-tryptone-based medium (CAT). Encapsulated *S. pneumoniae* serotype 2 D39 (NCTC7466)^26^ and TTL01 were cultured in Todd–Hewitt broth containing yeast extract (THY) or THY agar containing 5% (v/v) sheep blood as previously described^27^. The *Escherichia coli* strain was cultured in Luria broth (LB) medium. Optical densities at A_550_ or A_600_ were used to measure the growth of *S. pneumoniae* or *E. coli*, respectively.

**Table 1:** Bacterial strains and plasmids used in this study

Strains/plasmids	Relevant characteristics	Antibiotic resistance	Reference
Strains			
*S. pneumoniae*			
D39	Encapsulated, type 2		^26^
TTL01	D39 P_fcsK_::*adk* carrying an ermAM cassette	ERY	^3^
CP1200	Non-encapsulated derivative of Rx1 *malM511-str1*		^25^
TTL04	CP1200 P_fcsK_::*adk* carrying an ermAM cassette	ERY	^3^
R6	Non-encapsulated derivative of D39, no *cpsBCD*		^26^
*E. coli*			
BL21(DE3)	gal (cI ts857 ind1 Sam7 nin5 lacUV5-T7 gene 1)		Novagen
Rosetta2	Expresses seven rare tRNAs		Novagen
Plasmids			
pET32b	5899 bp; *E. coli* plasmid	AMP	Novagen
pCPS01	His-tagged *cps2B* in pET32b	AMP	This study
pCPS02	His-tagged *cps2C* in pET32b	AMP	This study
pGST-parallel 2	5038 bp; *E. coli* plasmid	AMP	^55^
pHis-parallel 2	5517 bp; *E. coli* plasmid	AMP	^55^
pGST-ADK	GST-tagged *SpAdK* in pGST-parallel 2	AMP	This study
pCPS03	His-tagged *cps2D* in pHis-parallel 2	AMP	This study
pCPS04	His-tagged *cps2D-3Y3E* in pHis-parallel 2	AMP	This study
pCPS05	His-tagged *cps2D-GA-KK* in pCPS03	AMP	This study
pCPS06	His-tagged *cps2D-GA-KK* in pCPS04	AMP	This study

*ERY* erythromycin, *AMP* ampicillin

Cloning and protein purification methods are described in detail in the Supplementary Information.

### Fucose-inducible conditional *adk* mutant strain construction

A conditional *adk* mutant controlled by a fucose promoter in either S (smooth, encapsulated/TTL01) or R (rough, non-encapsulated/TTL04) type strains (Table 1) was constructed by substituting the *adk* promoter with the P_*fcsK*_ inducible promoter in the pneumococcal chromosome to allow fucose regulation of SpAdK levels as described previously^3^.

### Preparation of total CPS

Total CPS was prepared from pneumococci grown in THY broth as previously described^28^. Pneumococci were harvested at *A*_550_ = 0.3 using centrifugation and rinsed twice with phosphate-buffered saline (PBS) before resuspending in PBS. The samples were calibrated to the same optical density at *A*_550_ to obtain the same concentration of bacteria. Subsequently, the pellets were resuspended in 500 μL of buffer A (Tris-Cl pH 7.0 and 1 mM MgSO_4_). The pellets were treated with 0.1% (w/v) sodium deoxycholate (DOC) at 37 °C for 15 min. Next, mutanolysin (100 U), DNase I (50 µg), and RNase A (50 µg) were added. The samples were slightly inverted at 37 °C for 18 h. Finally, the total CPS sample was treated with proteinase K (50 µg) and incubated at 56 °C for 4 h prior to storage at –20 °C. The samples were thawed on ice before the CPS quantification assay. All reagents used in this study were obtained from Sigma-Aldrich (St. Louis, MO, USA).

### Quantification of pneumococcal CPS by the phenol–sulfuric method

The amount of CPS was determined by a phenol–sulfuric assay. CPS samples were plated onto 96-well microplates, followed by addition of 150 µL of 95% H_2_SO_4_ (Duksan Pharmaceutical Co., Ansan, Korea) and 30 µL of 5% phenol (pH 8.2; Sigma-Aldrich). The mixture was incubated at 100 °C for 10 min, followed by cooling to 25 °C before measuring the optical density at *A*_490_. Because the *S. pneumoniae* serotype 2 CPS is composed of 2 U of d-glucose, 3 U of l-rhamnose, and 1 U of d-glucuronic acid^10^, a sugar solution of 2 U of d-glucose, 3 U of l-rhamnose, and 1 U of d-glucuronic acid (Sigma-Aldrich) was used as a standard control.

### Analysis of pneumococcal CPS using enzyme-linked immunosorbent assay (ELISA)

To measure total CPS, an indirect ELISA was performed with slight modifications as previously described^29^. *S. pneumoniae* was incubated in THY to *A*_550_ = 0.3. After centrifugation at 4000 × *g* for 10 min, the bacterial pellets were washed three times with PBS. The pellets from 1 mL of culture were suspended in 500 µL of PBS, and the optical density was adjusted to obtain similar amounts of bacteria. Next, the samples were heat killed at 65 °C for 20 min. After cooling to room temperature, 20 μL of heat-killed samples was coated onto plates containing 80 µL of PBS and immobilized for 4 h at 4 °C. The samples were further incubated with 1% (w/v) bovine serum albumin (Sigma-Aldrich) to block nonspecific binding. After washing with TPBS (PBS containing 0.05% Tween-20; Sigma-Aldrich), 100 μL of serotype 2 CPS rabbit antiserum (1:1000 in TPBS; Statens Serum Institut, Copenhagen, Denmark) was added to the samples and incubated at 25 °C for at least 90 min. Next, the plate was rinsed with TPBS three times. The samples were further incubated with secondary rabbit antibody conjugated to horseradish peroxidase (HRP; 1:10,000) at room temperature for 1 h. Next, the plate was washed with TPBS. The colorimetric substrate 3,3′,5,5′-tetramethylbenzidine (Biosciences BD, NJ, USA) was added to the wells, and *A*_650_ was measured using an ELISA reader (Softmax Pro 5.4).

### Western blotting

*S. pneumoniae* was grown exponentially in THY and lysed in lysis buffer (50 mM Tris-Cl, pH 8.0, 1 mM DTT, and 0.1% Triton X-100). Total protein was collected, run on a sodium dodecyl sulfate-polyacrylamide gel electrophoresis (SDS-PAGE) gel (10–20%), and transferred onto a 0.2-µm polyvinylidene difluoride (PVDF) membrane (Merck Millipore, MA, USA). The membrane was blocked with 3% (w/v) skim milk in TTBS (Tris-buffered saline [TBS] containing 0.1% Tween-20; Sigma-Aldrich) for 2 h at room temperature. Next, the membrane was probed overnight at 4 °C with polyclonal mouse sera raised against SpAdK, Cps2B, Cps2C, Cps2D, PsaA, and PspA appropriately diluted in TTBS. Next, the membrane was probed with HRP-conjugated anti-mouse immunoglobulin G (IgG) (1:10,000 in TTBS) for 1 h. The proteins were visualized by adding West-Q Pico ECL Solution (GeneDEPOT, TX, USA), captured using a Chemiluminescence Imaging System (Davinch-Chemi, Seoul, Korea), and analyzed using densitometry with the ImageJ software 1.4.3 (NIH, USA).

### Immunoprecipitation (IP)

IP and western blotting were performed as previously described^11^. Pneumococcal D39 WT lysate (100 µg; obtained as described for western blotting) was incubated with 5 µL of antibodies against SpAdK, Cps2D, or AdhE (negative control) at 4 °C for 4 h with gentle agitation. The mixture was mixed with 20 µL of Protein A/G PLUS Agarose (Santa Cruz Biotech, CA, USA) and gently agitated overnight at 4 °C. The beads were collected and washed six times in lysis buffer. IP proteins were detached from the beads by adding 50 µL of 2 × SDS-PAGE loading buffer and boiled at 100 °C for 4 min. The supernatant (20 µL) was loaded onto 15% SDS-PAGE gels and analyzed using western blotting with SpAdK, CpsD, or AdhE antibodies.

### Bio-layer interferometry (BLI)

BLI experiments were performed using a Blitz system (ForteBio, CA, USA). His_6_-Cps2D WT or 3Y3E mutant protein (1 μM) were immobilized on Ni-NTA sensors (ForteBio, CA, USA). Binding of SpAdK was monitored by adding various concentrations of glutathione *S*-transferase (GST)-SpAdK (2.5–50 μM) in buffer D (250 mM NaCl, 50 mM Tris-HCl, pH 7.5, and 1 mM DTT) in the absence or presence of 5 mM Mg-ATP. BLI experiments with Cps2D GK-AA and GK-AA/3Y3E were similarly performed in buffer D. GST protein was used as a negative control, and background signals from GST and sensors were subtracted before binding analysis. The experiments were repeated at least twice. *K*_d_ values were determined using steady-state analysis with GraphPad Prism (GraphPad Software Inc, CA, USA).

### Fluorescence microscopy

For visualization of fluorescein isothiocyanate (FITC)-labeled CPS, *S. pneumoniae* was grown exponentially in THY broth, and bacteria were collected by centrifugation. Subsequently, the cells were fixed with 5% formaldehyde (Sigma-Aldrich) by incubating for 15 min, rinsed twice with distilled water, and resuspended in 0.1 volume of the original culture volume of PBS. The suspension (100 µL) was cross-reacted with the type 2 rabbit antiserum (1:500 in PBS; Statens Serum Institut) for 90 min and probed with anti-rabbit FITC (1:1000 in PBS; Sigma-Aldrich) for 1 h. Samples (10 µL) were mounted on a slide, covered with 50% glycerol, and visualized using a confocal microscope (LSM 510; Carl Zeiss; Oberkochen, Germany).

### Phos-tag^TM^ acrylamide gel and western blotting

*S. pneumoniae* was grown exponentially in THY and lysed in lysis buffer. Total protein was collected and run on a Phos-tag^TM^ acrylamide gel (10% SDS-PAGE supplemented with 25 µM Phos-tag^TM^ AAL-107 [Wako Pure Chemical, Osaka, Japan] and 100 µM MnCl_2_ [Sigma-Aldrich]). Next, the gel was soaked in transfer buffer with 1 mM EDTA with light agitation for 10 min to eliminate Mn^2+^ from the gel. Next, the gel was briefly rinsed with transfer buffer without EDTA once and re-soaked in transfer buffer (no EDTA). The proteins were transferred from the gel to a PVDF membrane (Merck Millipore). After transfer, the membrane was blocked with 3% (w/v) skim milk (Merck) in TTBS for 1 h at room temperature. The membrane was washed with TTBS for 30 min and probed with total Cps2D antibody (1:1000 dilution in TTBS) overnight at 4 °C. The membrane was again washed with TTBS for 40 min and probed with HRP-conjugated anti-mouse IgG (1:10,000 in TTBS) for 1 h. The proteins were visualized by adding West-Q Pico ECL Solution, captured using a Chemiluminescence Imaging System, and analyzed using densitometry with ImageJ software.

### Sequence analysis and structural modeling of the SpAdK and Cps2D proteins

Cps2D is a tyrosine kinase of approximately 226 amino acids and no three-dimensional (3D) structural information to date. To predict the 3D structure of full-length Cps2D protein (UniProtKB ID: Q9ZII6), we utilized MOE, I-TASSER^30^, and SwissModel servers. These packages predicted similarly folded structures for Cps2D except for the C-terminus flexible tyrosine-rich region. Based on sequence identity and coverage, the C-terminus was not properly predicted by MOE and SwissModel. However, the models provided by I-TASSER (using a threading technique) were properly folded and retained the C-terminus tail, which is necessary for Cps2D phosphorylation. The structure of SpAdK was previously studied in open and closed conformations using X-ray crystallography (PDB ID: 4NU0, 4NTZ). Both conformers were retrieved from PDB and prepared for docking analyses.

### Docking simulations

After thoroughly investigating the structure of Cps2D, ATP, and ADP were docked into the active site suggested by I-TASSER and confirmed through sequence and structural alignments with other bacterial-encoded tyrosine kinases. The induced fit docking method implemented in MOE was used to predict the binding ability and affinity of phospho-adenosine to the active site of Cps2D. Before docking, the predicted model was optimized using the protein preparation tool in MOE. Partial charges and hydrogens were added where needed. The structure was relaxed and energy minimized to remove steric clashes, keeping the backbone atoms fixed and the side-chains flexible using Amber10: EHT force field. The ligands were docked into the suggested active site using the default induced fit docking protocol in MOE.

To evaluate the possible mechanism of Cps2D tyrosine phosphorylation and its interaction with SpAdK, we divided the protein–protein docking simulation into two steps. First, to understand the autophosphorylation mechanism, Cps2D was docked with itself using ZDOCK^31^, PatchDock^32^ online server, and MOE. Second, to understand the binding mechanism of Cps2D and SpAdK as suggested by our empirical results, they were docked using the same protocols. The docked conformers provided by ZDOCK and PatchDock were submitted to FireDock for refinement^33^. FireDock simultaneously targets the problem of flexibility and scoring of solutions produced by the fast, rigid body docking algorithms of PatchDock and other servers. FireDock refines and scores a large set of complexes selected from input files and sorts them according to their minimum global energy. Interface analyses were performed in MOE, UCSF Chimera, and PyMol^34^, whereas images were generated in MOE and PyMol. Structure and sequence alignments and comparisons were performed in MOE and BioEdit.

### Statistical analysis

Statistical analysis between the different experimental groups in the phenol–sulfuric acid assay, ELISA, western blotting, adherence, survival, and phagocytic activity assays were calculated using a one-way analysis of variance (ANOVA) (Duncan’s method, non-parametric). ELISA data are expressed as the mean ± standard deviation (SD; *n* = 3). A non-parametric, two-tailed value of *P* *≤* 0.05 (*), *P* *≤* 0.01 (**), or *P* *≤* 0.001 (***) was considered statistically significant.

## Results

### Increase in pneumococcal CPS levels by SpAdK

**Fig. 1:**
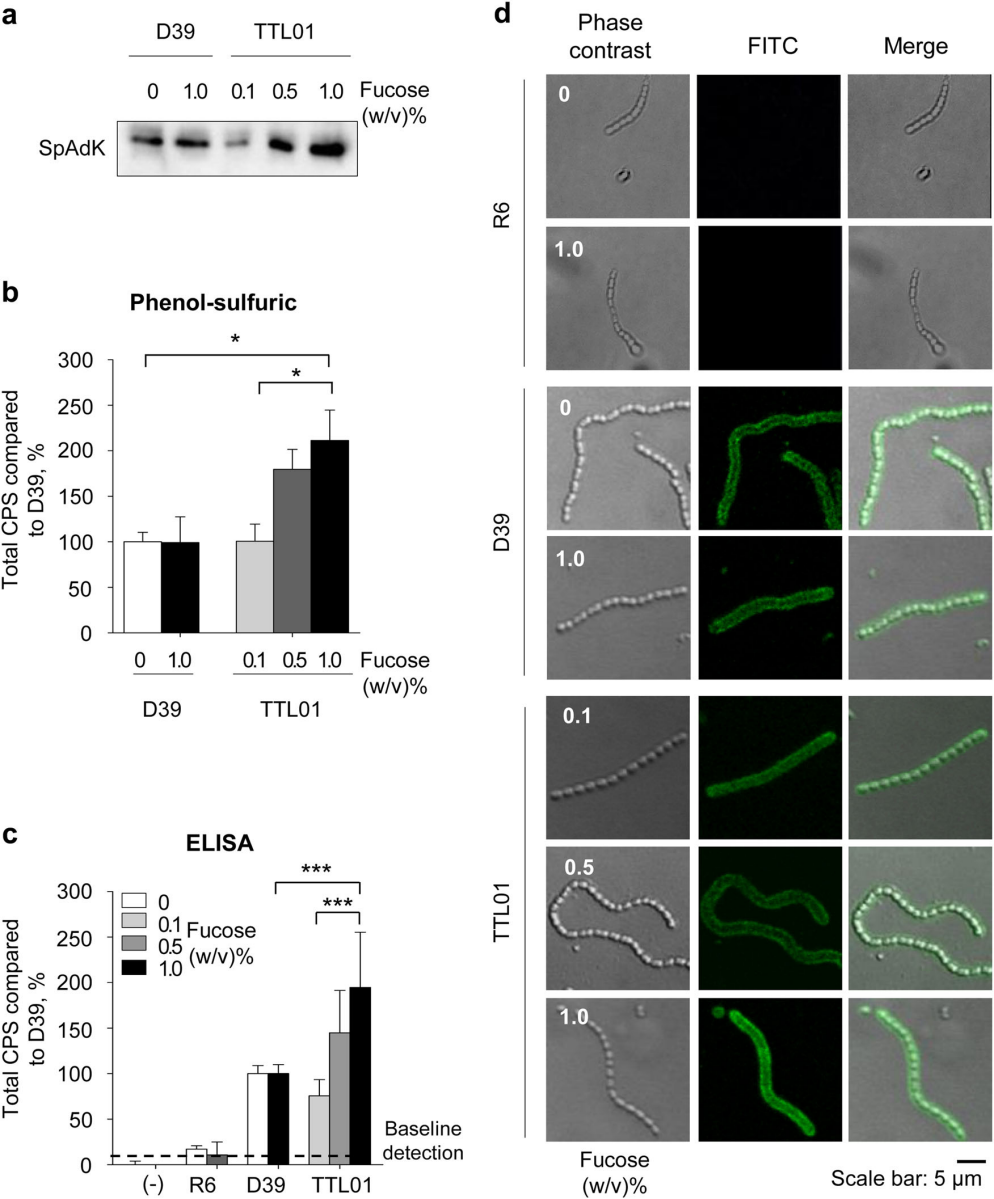
SpAdK expression increases capsular polysaccharide (CPS) levels. **a** Protein levels of SpAdK from lysates of *S. pneumoniae* D39 WT and TTL01 strains under various concentrations of fucose. **b**, **c** Total CPS from whole-cell lysates of *S. pneumoniae* D39 WT and TTL01 strains under various concentrations of fucose was isolated, and the amount was measured using (**b**) the phenol–sulfuric method and **c** ELISA after cross-reacting with the type 2 CPS antiserum. All samples were assayed in triplicate, and each assay was repeated three times. Significant differences were analyzed by one-way ANOVA (*P ≤ 0.05 and ***P ≤ 0.001). **d** FITC-labeled CPS of *S. pneumoniae* was visualized by confocal microscopy. The data are representative of three independent experiments

SpAdK was previously shown to be essential for pneumococcal growth. To confirm that SpAdK expression is essential for CPS synthesis, we used the encapsulated TTL01 strain, which is under the control of a fucose promoter^3^, and analyzed SpAdK expression and CPS levels under various concentrations of fucose (0, 0.1, 0.5, and 1.0%). Fucose can control SpAdK levels in the TTL01 strain but not in D39 WT (Fig. 1a), indicating that fucose does not affect D39 WT SpAdK levels. These results agreed with our previous findings, suggesting that differences in conditional *adk* mutant strains were due to different SpAdK levels^3^.

To quantify CPS levels, the phenol–sulfuric assay showed that total CPS levels of TTL01 significantly increased at 1.0% compared with 0.1% fucose (Fig. 1b). Consistently, ELISA results also showed a substantial increase in TTL01 CPS levels after supplementing with 1.0% fucose compared with 0.1% fucose-treated cells (Fig. 1c). These findings indicate that CPS levels were significantly increased in the TTL01 strain after treating with 1% fucose compared with WT (Figs. 1b, c). The D39-derived non-encapsulated *S. pneumoniae* R6 strain (*∆cpsBCD*) was used as a negative control in this experiment. Almost no CPS was detected in R6 samples regardless of fucose concentration (Fig. 1c). To exclude the possibility of fucose affecting CPS quantitation assays, D39 WT was incubated with various concentrations of fucose (0, 0.1, 0.5, and 1.0%) and CPS levels were measured. We found that fucose did not show any significant effects on CPS levels (data not shown).

To confirm the dependence of CPS on SpAdK levels, the R6, D39 WT, or TTL01 strains were grown to log phase in varying concentrations of fucose. Next, the harvested bacterial pellets were treated with CPS antibody and visualized using confocal microscopy. The TTL01 strain at 1.0% fucose showed stronger fluorescence than 0.1% fucose, but D39 WT did not show any significant difference in fluorescence intensity after fucose supplementation (Fig. 1d). We used non-encapsulated *S. pneumoniae* R6 as a negative control and almost no FITC-CPS signal was detected at any level of fucose.

### SpAdK increases Cps2D levels

**Fig. 2:**
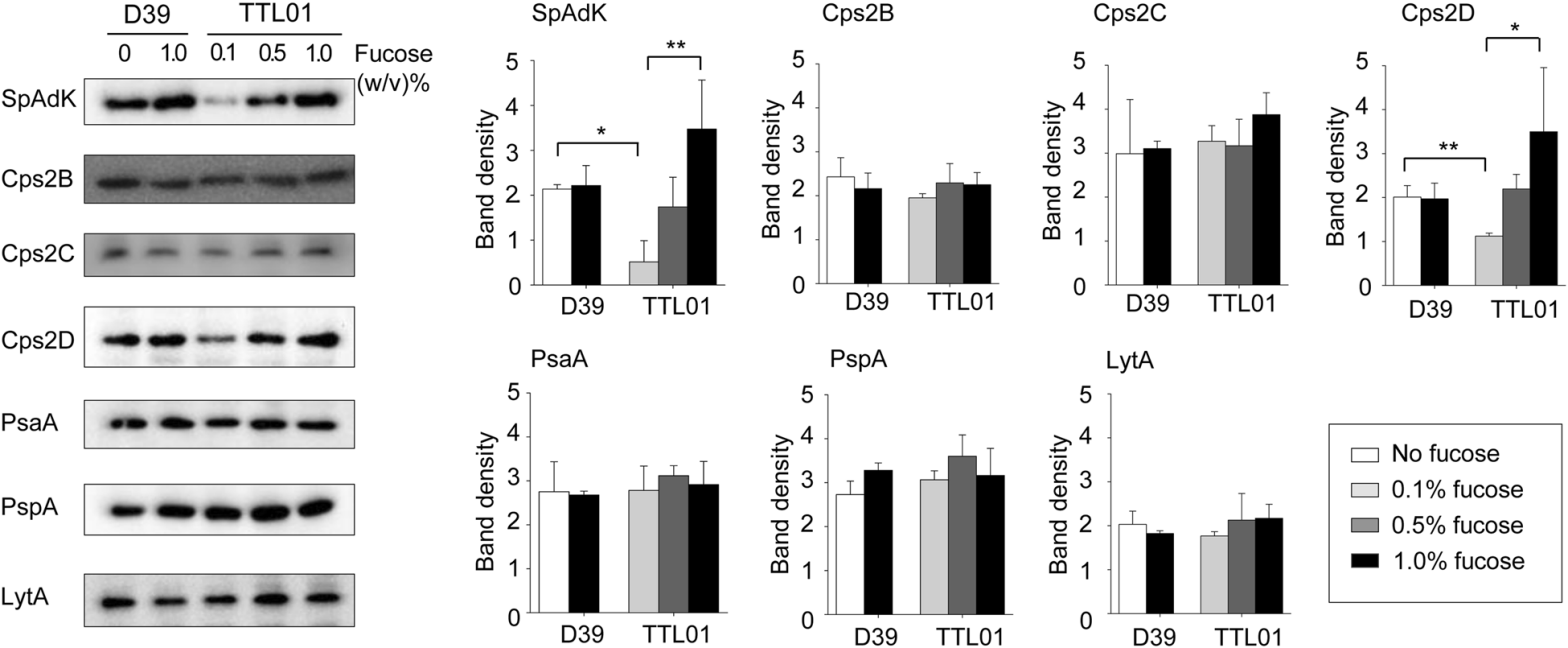
SpAdK increases Cps2D but not Cps2B/C levels. *S. pneumoniae* lysates were collected and subjected to western blotting with appropriate antibodies. The experiments were repeated independently three times. ImageJ software was used for densitometry analysis and to determine statistical analysis. Significant differences were analyzed using one-way ANOVA (*P ≤ 0.05 **P *≤* between 0.05 and 0.001 and ***P ≤ 0.001)

The autophosphorylating tyrosine kinase Cps2D supplies ATPs that provide energy during CPS synthesis^10,35^. SpAdK regulates ATP levels in *S. pneumoniae*^3^ and increases CPS levels. However, the underlying mechanism remains unclear. To elucidate SpAdK-mediated *cps* locus regulation, the expression profiles of Cps2B, Cps2C, and Cps2D were compared between the D39 WT and TTL01 strains after fucose treatment using specific antibodies raised against these proteins (Supplementary Information). In the TTL01 strain, expression levels of Cps2B and Cps2C did not change, but Cps2D levels increased significantly along with SpAdK levels (Fig. 2). Additionally, fucose did not affect Cps2B, Cps2C, and Cps2D levels in D39 WT (Fig. 2). Moreover, SpAdK did not affect autolysin (LytA) and surface protein (PsaA and PspA) expression levels in both D39 WT and TTL01 strains (Fig. 2).

### SpAdK enhances Cps2D phosphorylation

**Fig. 3:**
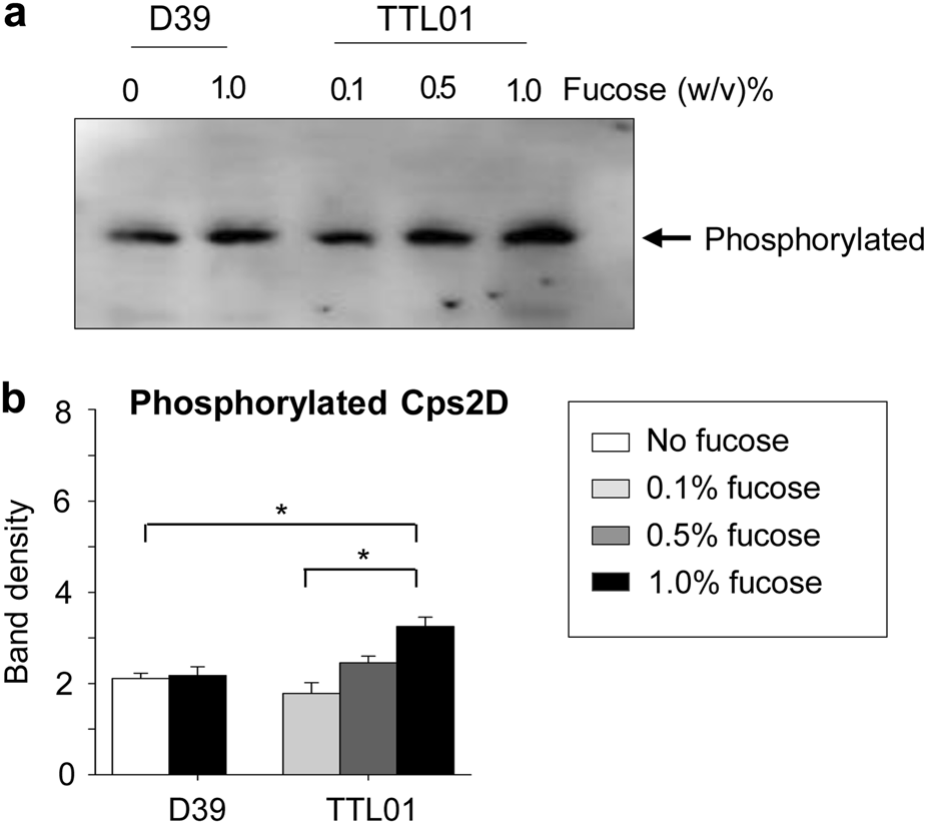
SpAdK increases phosphorylation of Cps2D. **a** Pneumococcal lysates were subjected to Phos-tag^TM^ SDS-PAGE and western blotting with anti-Cps2D to compare phosphorylation levels of Cps2D between *S. pneumoniae* D39 WT and TTL01 strains. The data are representative of three independent experiments. **b** ImageJ was used for densitometry analysis and to determine the differences in phosphorylated Cps2D levels between D39 WT and TTL01 strains. One-way ANOVA was used for statistical analysis (*P *≤* 0.05 and **P *≤* 0.01)

Cps2D contains an ATP-binding domain and requires ATP to autophosphorylate its C-terminus tyrosine cluster^35^. Moreover, the increase in Cps2D levels is directly proportional to SpAdK levels (Fig. 2). To address the effect of SpAdK on the phosphorylation of Cps2D, Phos-tag^TM^ SDS-PAGE was used to determine phosphorylated Cps2D levels, because the Phos-tag^TM^ complexed with Mn^2+^ can detect phosphorylated proteins in SDS-PAGE^36^. After densitometry analysis, the phosphorylated Cps2D protein was found to increase proportionally with SpAdK levels in the TTL01 strain (Fig. 3a, b). Moreover, phosphorylated Cps2D levels in TTL01 after 1% fucose treatment were significantly higher than in D39 WT (Fig. 3a, b). To test whether fucose affects protein levels, D39 WT was incubated with the highest fucose concentration (1.0%). However, 1.0% fucose did not affect the Cps2D protein level. Thus, the difference in proteins levels was due to SpAdK levels and not fucose. These results indicate that the interaction between SpAdK and the Cps2D proteins enhanced phosphorylation of Cps2D and resulted in increased CPS levels.

### SpAdK physically interacts with Cps2D

**Fig. 4:**
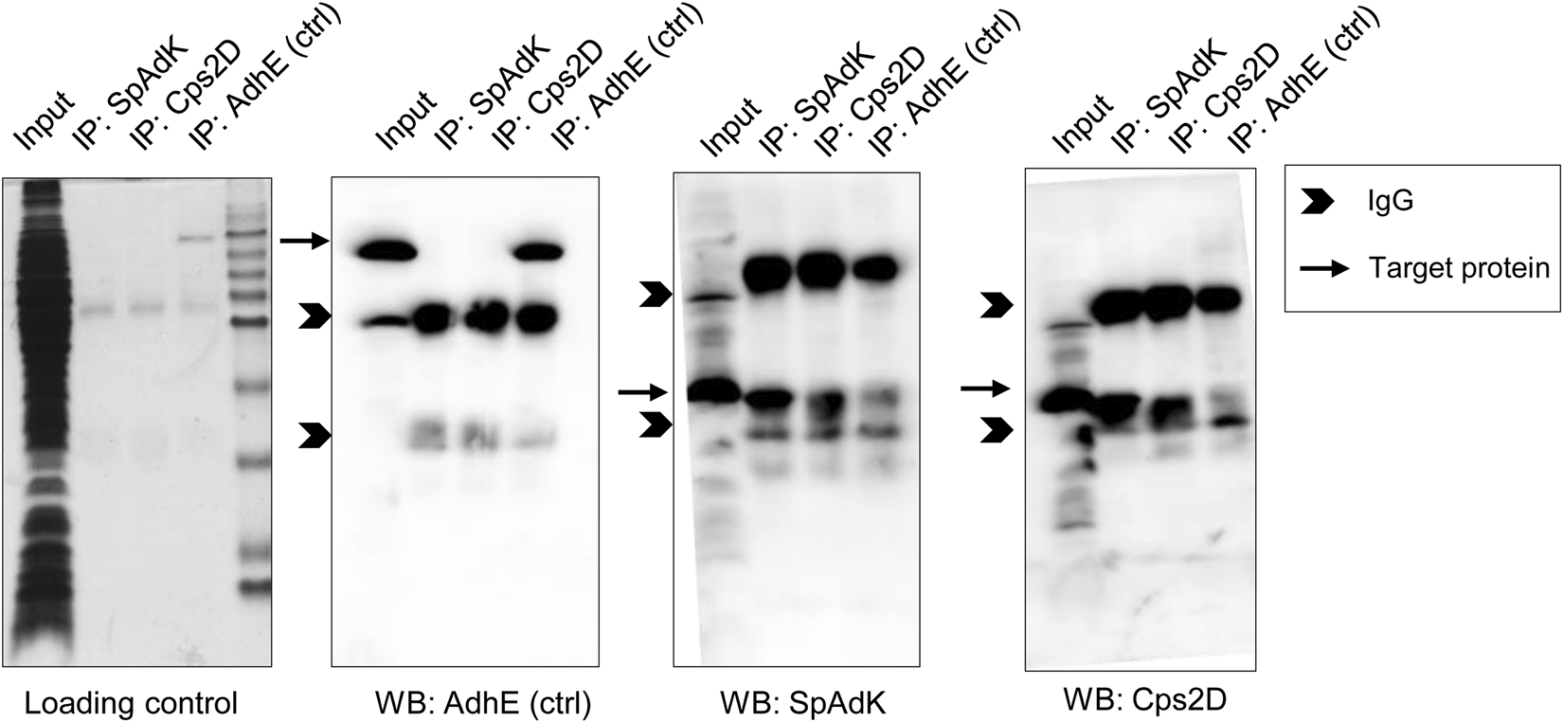
SpAdK interacts with Cps2D. Lysates (100 µg) were used for immunoprecipitation with 5 µL of anti-SpAdK, anti-Cps2D, or anti-AdhE (control). Immunoprecipitated proteins were subjected to western blotting. For western blotting, proteins were detected with anti-SpAdK, anti-Cps2D, or anti-AdhE

As SpAdK modulates Cps2D levels, we analyzed whether SpAdK interacts with Cps2D using an IP assay with antibodies against SpAdK, Cps2D, and AdhE (as a negative control), followed by western blotting. IP results showed that SpAdK physically interacts with Cps2D (Fig. 4). The AdhE antibody did not show any interaction with SpAdK and Cps2D (Fig. 4). Moreover, the IgG proteins were always detected in the IP studies, including SpAdK, Cps2D, and AdhE (control). However, the target protein was detected only for IP: SpAdK and IP: Cps2D samples, indicating a physical interaction between SpAdK and Cps2D.

### Biophysical characterization of the SpAdK–Cps2D complex

**Fig. 5:**
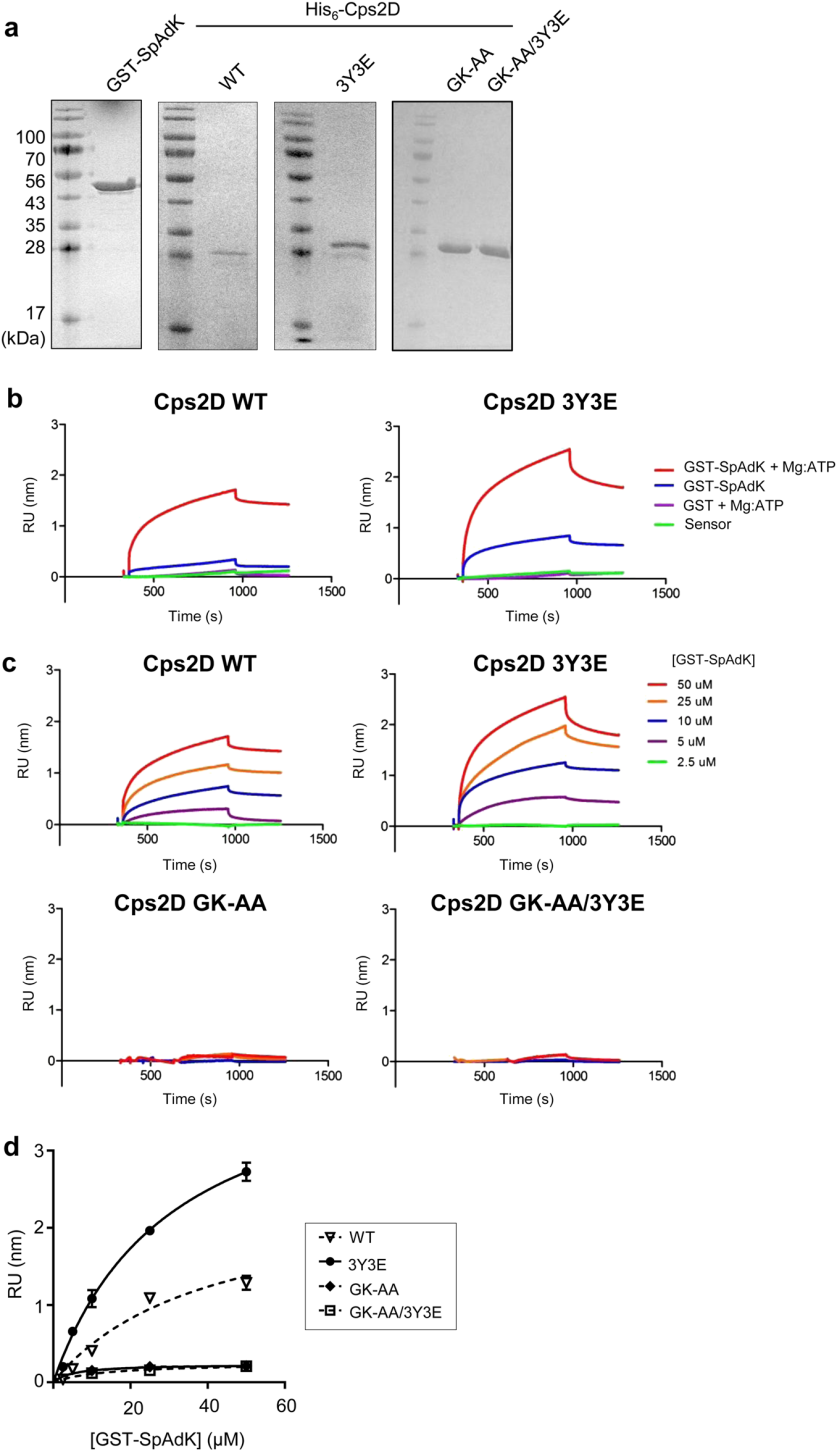
Cps2D ATP-binding domain is required for direct interaction with SpAdK. **a** SDS-PAGE gels showing purified recombinant GST-SpAdK, His_6_-Cps2D wild-type (WT), 3Y3E (Y215E/Y218E/Y221E), GK-AA (G48A/K49A), and GK-AA/3Y3E. **b** BLI sensorgrams of the interactions between SpAdK and Cps2D WT or 3Y3E in the presence or absence of ATP. For clarity, only sensorgrams at the maximal concentration of Cps2D protein (50 μM) are shown. **c** BLI sensorgrams of the interaction between SpAdK with Cps2D. His_6_-Cps2D was immobilized on Ni-NTA sensors, followed by the addition of varying concentrations of GST-SpAdK (2.5, 5, 10, 25, and 50 μM). RU response unit. **d** BLI-derived binding curves for the interactions of SpAdK with Cps2D WT, 3Y3E, GK-AA, and GK-AA/3Y3E mutants for steady-state binding analysis. The data are representative of two independent experiments

Mutating tyrosine (Y) to glutamic acid (E) in the C-terminus tyrosine cluster was previously used in in vitro and in vivo studies to determine protein phosphorylation^37,38^. To investigate the effect of Cps2D phosphorylation on its interactions with SpAdK, we prepared a triple phosphomimetic mutant, 3Y3E, where three tyrosine residues (Y215, Y218, and Y221) in the tyrosine cluster of Cps2D were mutated to glutamic acid. The WT and the 3Y3E mutant of Cps2D were purified to homogeneity (Fig. 5a). Quantitative binding analysis using BLI revealed that SpAdK binds to both WT and 3Y3E in the presence of Mg-ATP with apparent *K*_d_ values of 31 ± 5 μM and 40 ± 17 μM, respectively (Fig. 5b-d).

The ATP-binding domain of Cps2D contains conserved glycine (G) 48 and lysine (K) 49 amino-acid residues that are shared with other bacteria^35^. To determine whether the ATP-binding domain of Cps2D plays any role in the interaction with SpAdK, we prepared double mutants of G48 and K49 mutated to alanine (A) in the background of Cps2D/WT and Cps2D/3Y3E termed GK-AA and GK-AA/3Y3E, respectively. SpAdK did not bind to either GK-AA or GK-AA/3Y3E, suggesting that ATP-binding residues have a crucial role in the SpAdK–Cps2D interaction (Fig. 5c, d). These results show that phosphorylation of the tyrosine cluster of Cps2D does not interfere with its interaction with SpAdK, whereas the ATP-binding capability of Cps2D is critical in mediating the interaction between Cps2D and SpAdK.

### In silico structural analysis of Cps2D and the Cps2D–SpAdK interaction mechanism

**Fig. 6:**
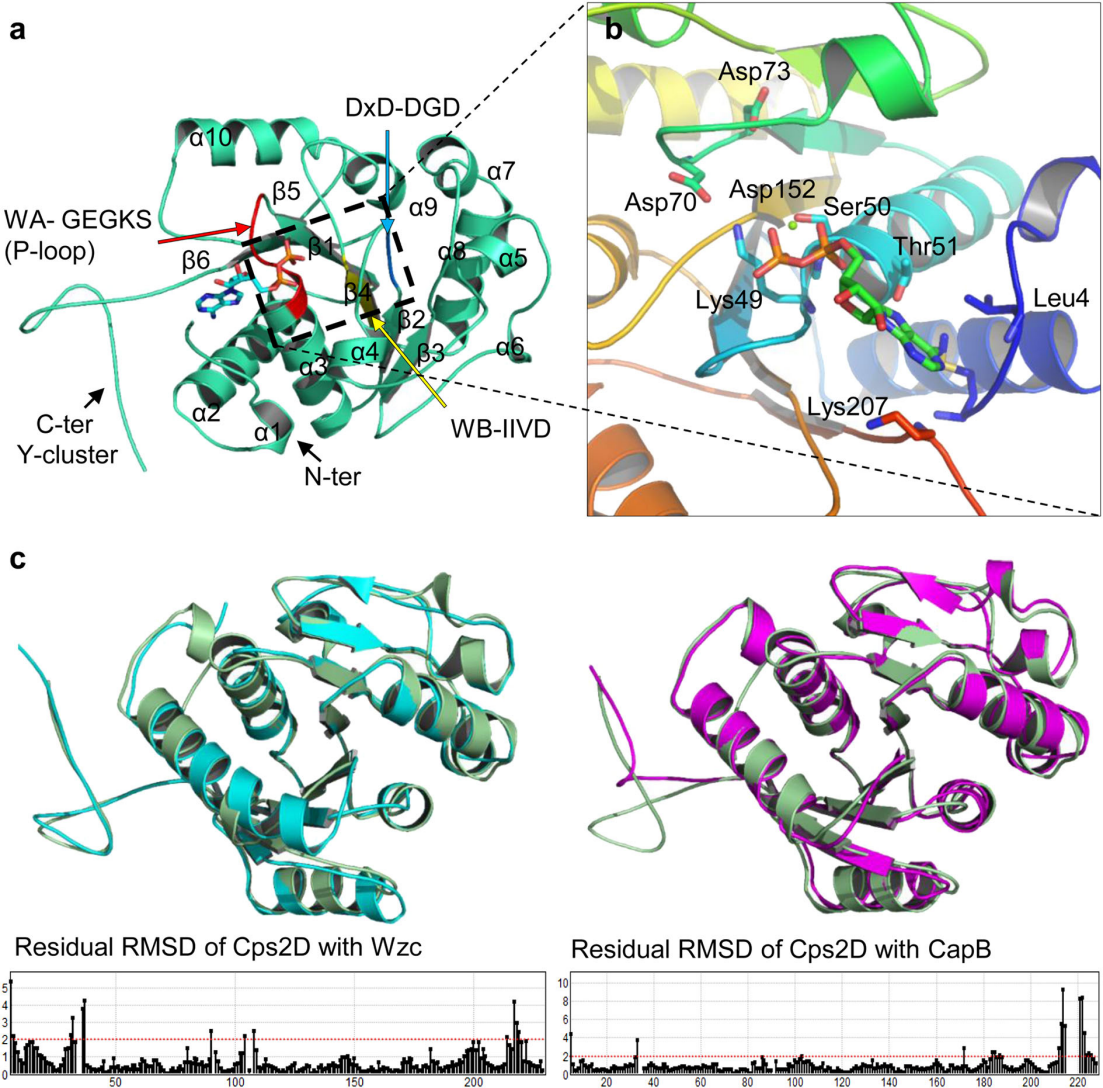
Structural analysis of Cps2D. **a** 3D model of Cps2D exhibiting typical BY-kinase-like structure. The red loop highlights the Walker A (WA) motif containing ATP-binding conserved residues and the p-loop (α2–β1). The cyan color indicates the catalytic DxD motif, and the yellow color marks the Walker B (WB) motif. These motifs are essential for the enzymatic activity of tyrosine kinases. **b** The ADP-bound active site of Cps2D with the conserved L49 and DxD catalytic domain. **c** Our Cps2D model superimposed over previously reported crystal structures of tyrosine kinase from *E. coli* (Wzc) and *S. aureus* (CapB)

Based on our empirical results, we proposed a structural model that describes autophosphorylation of Cps2D and its interaction with SpAdK. The optimized 3D structure of Cps2D is a correctly folded protein that shares domain organization with the previously reported BY-kinases (bacterial tyrosine kinases), CapB^39^ and Wzc^40^. Structural and sequence alignment confirmed that Cps2D is a P-loop-containing protein kinase with Walker A^41^ nucleotide-binding motifs in its core region. The loop between α3 and β1 containing –GEGKS– residues corresponds to the defined P-loop, which is involved in ATP binding (Fig. 6a). This loop establishes typical interactions with docked ADP. Similar results were previously reported^39^. The interaction between ADP and bound Mg^2+^ ions is further stabilized by the DxD motif (containing aspartic acid [D] 70 and aspartic acid [D] 73, located 20 amino acids downstream of the Walker A motif) and D152 of the Walker B motif (Fig. 6b). Structural alignment suggested that Cps2D shares domain organization and sequence identity with *E. coli* Wzc (RMSD 1.02) more than the *Staphylococcus aureus* CapB kinase (RMSD 1.5; Fig. 6c).

In this study, we reported that SpAdK establishes direct contact with Cps2D and enhances its phosphorylation levels. To further elucidate this mechanism, we proposed a possible interaction mechanism between SpAdK and the Cps2D dimer bound to ATP. It was reported that the C-terminus of *S. aureus* CapA acts as an activator for the CapB tyrosine kinase and exists in the chimeric “CapA/CapB” form^39^. We also found that WT SpAdK and its triple mutant 3Y3E interact with purified Cps2D, suggesting a similar role for the enzymatic activity of Cps2D as CapA in CapA/CapB. However, there are some discrepancies that cannot be explained by our trimeric complex.

**Fig. 7:**
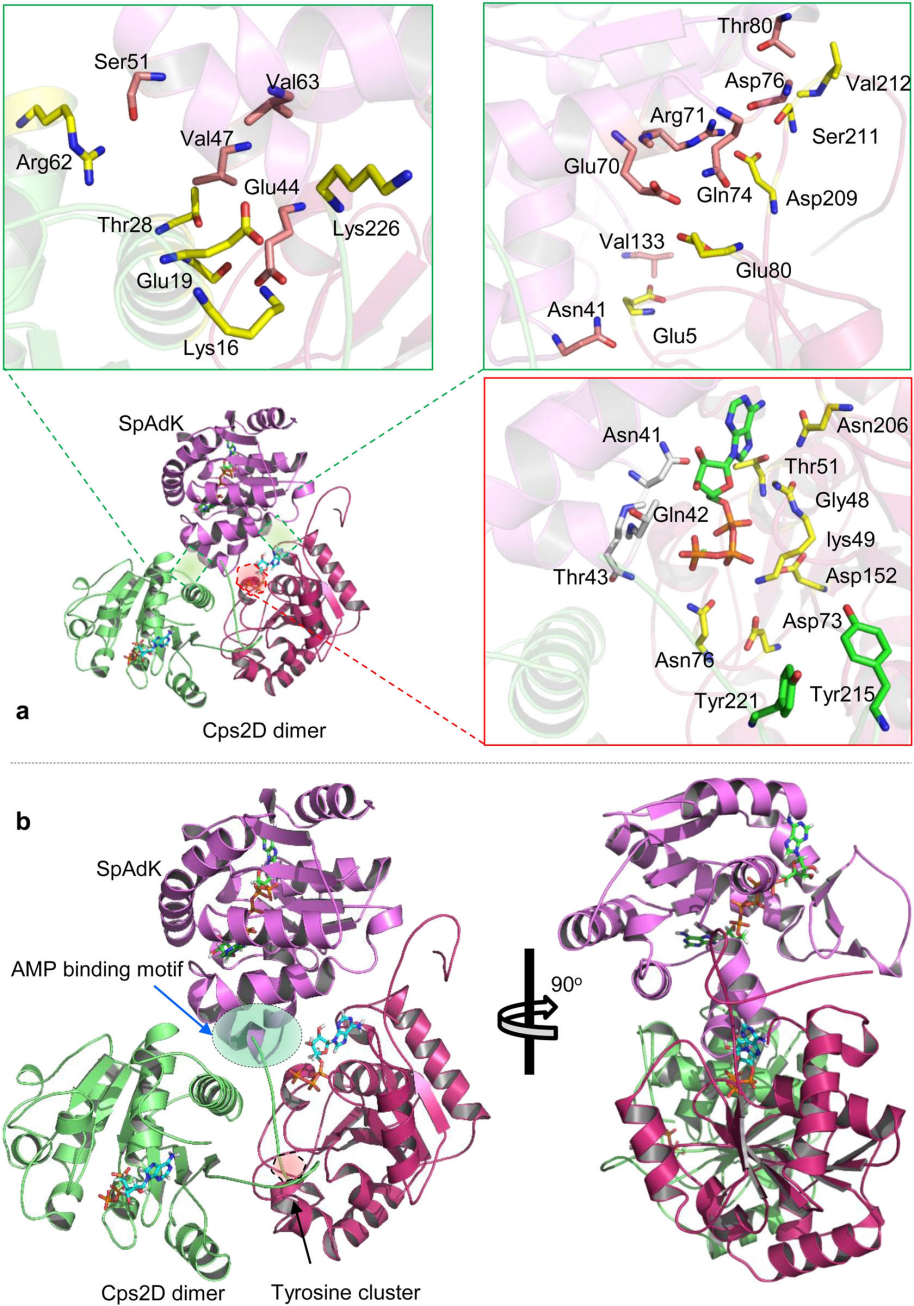
The binding mechanism of Cps2D and SpAdK. **a** The binding interface of the trimeric (Cps2D)_2_SpAdK. Green boxes highlight the protein interface of SpAdK with each monomeric Cps2D. The red boxes illustrate the role of ATP and the ATP-binding interface in the Cps2D–SpAdK interaction and the autophosphorylation mechanism. **b** Front and lateral views of the (Cps2D)_2_SpAdK complex. The red shaded area highlights the C-terminus tyrosine cluster bound to the active site of Cps2D, whereas the green shaded area illustrates the “V”-shaped AMP-binding motif of SpAdK anchoring oligomeric Cps2D

Overall, the proposed model suggests that SpAdK stabilizes the interface between oligomeric Cps2D. Charged and uncharged polar residues are mainly involved at the heterotrimeric interface (Fig. 7a). The “V”-shaped helical motif of SpAdK that anchors the two Cps2D dimers was reported to be involved in AMP binding^3^. As SpAdK regulates ATP levels, we theorize that P_i_ released through this motif may possibly be involved in the phosphorylation of Cps2D. Moreover, the polar residues in this motif establish direct contact with ATP and its binding interface on one of the bound Cps2D monomers (Fig. 7b).

## Discussion

AdK reversibly catalyzes the nucleotide phosphoryl exchange reaction^17^. The role of AdK in the regulation of adenine nucleotide homeostasis is well established^17^. Pyruvate oxidase has a direct influence on ATP production and is a crucial link between pneumococcal metabolism and CPS synthesis^18^. In this study, we report for the first time that pneumococcal capsule synthesis is directly regulated by SpAdK through its interaction with the Cps2D protein. Thus, the amount of ATP required for CPS synthesis in the encapsulated *adk* mutant strain might retard growth compared with the non-encapsulated strain (Supplementary Figure S1).

Tyrosine kinases are crucial for CPS regulation^42,43^. For instance, a mutant of the tyrosine kinase, Wzc, of the Gram-negative *E. coli* serotype K30 impairs the production levels of its high-molecular-weight capsule^44^. Furthermore, the ExoP mutant of *Sinorhizobium meliloti* produces less succinoglycan (of the exopolysaccharide) by altering ATPase activity^45^. The CpsD of *S. pneumoniae* is comparable to the Wzc, CapB, and YwqD proteins present in *E. coli, S. aureus*, and *Bacillus subtilis*, respectively^46^. Cps2D is essential for CPS synthesis, and a *cps2D* deletion mutant showed reduced CPS levels^14^. Pneumococcal capsule synthesis is driven by the ATP generated by Cps2D, whereas SpAdK regulates ATP levels in *S. pneumoniae*. Therefore, there may be a possibility that SpAdK increases Cps2D levels and ultimately results in increased ATP-mediated CPS synthesis. Our results indicate that increased SpAdK levels led to a significant increase in total and phosphorylated Cps2D levels, which eventually resulted in enhanced CPS production (Figs. 1, 2, and 3). A previous report showed that the deletion of cps2B resulted in increased tyrosine-phosphorylated Cps2D levels that enhanced CPS production levels in *S. pneumoniae* D39^14^. This is consistent with a study by Weiser et al.^47^ that showed that reduced environmental oxygen levels resulted in increased pneumococcal capsule production via increased phosphorylation of Cps2D. Although a recent study suggested that the levels of Cps2D phosphorylation are not a determinant for CPS regulation, they did not exclude the role of Cps2D in CPS synthesis^48^. However, it was also reported that autophosphorylation of Cps2D negatively influences pneumococcal CPS production^6^. To date, an underlying mechanism that resolves these conflicting results remains to be elucidated.

CPS is required for asymptomatic pneumococcal colonization of the respiratory tract or for invasive diseases^12^. During colonization, *S. pneumoniae* expresses low levels of CPS (transparent type) to enhance the exposure of cell surface proteins and promote binding to epithelial cells^49^. During high oxygen availability, *S. pneumoniae* D39 reduces Cps2D phosphorylation, resulting in reduced CPS levels^48^. In contrast, *S. pneumoniae* expresses high CPS levels (opaque type) during systemic infection to evade complement-mediated opsonophagocytosis^12^. *S. pneumoniae* serotype 6A opaque variants show enhanced Cps6aD expression compared with transparent variants^47^. Therefore, survival of *S. pneumoniae* throughout various invasive steps requires modulation of CPS expression to adapt to different niches. How pneumococci manipulate capsular gene expression levels between colonization (thin capsule) and invasion (thick capsule) remains unclear. SpAdK expression was shown to be induced by serum^3^. In addition, our results show that SpAdK increases CPS levels by upregulating Cps2D. Accordingly, transcript levels of the *cps* locus were also elevated by SpAdk (Supplementary Figure S2). Pneumococci present in the bloodstream produce a thick capsule. Thus, pneumococci may induce expression of specific enzymes that subsequently increase ATP levels and CPS synthesis. This warrants further investigation of whether SpAdK is produced by pneumococci in the blood to supply ATP for energy metabolism and CPS synthesis.

CPS is known to inhibit adherence to host cells^50^ and protect bacteria from the host immune system. *S. pneumoniae* with a thin capsule show higher levels of cell wall phosphorylcholine and surface proteins, which allows stronger binding to epithelial cells^51,52^. Adherence of the TTL01 strain to the macrophage RAW 264.7 and HEp-2 cells significantly decreased proportionally to SpAdK levels (Supplementary Figure S3). Additionally, protein levels of PsaA and PspA in the TTL01 strain did not show any significant difference from D39 WT (Fig. 2). Therefore, SpAdK appears to affect multiple factors by increasing the surface energy charge of pneumococci and CPS levels that may play important roles in adherence. CPS also inhibits phagocytosis^53^ because CPS expression increases resistance to complement C3 deposition and subsequently to phagocytosis by neutrophils^51,54^. To confirm the dependence of CPS on SpAdK, the effect of SpAdK on the phagocytic activity of macrophages was analyzed. Phagocytosis was inhibited at high SpAdK expression levels and high CPS levels (Supplementary Figure S4).

To explore the underlying mechanism behind SpAdK-mediated CPS regulation, we performed IP assays to assess if SpAdK directly interacts with Cps2D. Our results showed a physical interaction between SpAdK and Cps2D (Fig. 4). However, as the molecular sizes of SpAdK (approximately 27 kDa) and Cps2D (27 kDa) are quite close to that of the IgG light chain (25 kDa), we could not get satisfactory IP results with western blotting even after repeated efforts (Fig. 4). To gain insights into the SpAdK–Cps2D interaction and to further confirm our IP data, we performed a BLI experiment with purified proteins. This showed that SpAdK can interact with the Cps2D protein in the presence of Mg-ATP (Fig. 5). However, it was not necessary to add exogenous Mg-ATP during the IP assay to support the interaction of SpAdK and Cps2D because pneumococcal cell lysate, a great source of Mg-ATP, was used. Moreover, the importance of the ATP-binding domain of Cps2D in this interaction was also established.

To model the signaling mechanisms of proteins in a protein network, a molecular docking study was utilized to predict the preferred orientation of the two interacting chemical species. The in silico structural similarities and domain organizations indicated that, like other BY-kinases, Cps2D may have a similar role in CPS production (Fig. 6), which agrees with previous findings. In line with the previously reported autophosphorylation mechanism of BY-kinases, our model also suggests a similar mechanism (Supplementary Figure S5). The tyrosine-rich motif located at the distant C-terminus, which is involved in autophosphorylation, fits near the ATP-binding site of the adjacent Cps2D. The proposed model is in line with our mutational and biophysical analyses, which showed that mutating the conserved ATP-binding residues, G48 and K49, abolished the Cps2D–SpAdK interaction (Fig. 7). As the BLI analyses were performed in the presence of Mg-ATP, mutating the two conserved residues dismantled the ATP interaction and dissociated the interaction with SpAdK. BLI analyses suggest that Cps2D binds SpAdK in the presence of Mg-ATP regardless of the presence or absence of the C-terminus tyrosine cluster. This was supported by our model, which suggests that Cps2D does not interact with SpAdK through its C-terminus. Cumulatively, these results provide a mechanism of the Cps2D-ATP/ADP binding, Cps2D autophosphorylation, and the role of SpAdK in the regulation of Cps2D.

Based on their regulatory role in polysaccharide production, BY-kinases were classified as polysaccharide co-polymerases, supporting the polymerase function of the membrane polysaccharide assembly machinery. However, the in-depth mechanism by which BY-kinases interact with this machinery remains unclear. Our study may provide a significant contribution to understanding the underlying mechanism behind BY-kinase-mediated regulation of capsule production.

Altogether, our findings indicate that cellular ATP and total CPS levels increase proportionally to SpAdK levels, establishing the involvement of SpAdK in CPS production. Furthermore, the involvement of SpAdK in CPS synthesis appears to be mediated by Cps2D phosphorylation, a key regulator in the CPS biosynthesis pathway. Based on the significance of CPS in pneumococcal systemic virulence, it would be a sensible approach to develop small molecular inhibitors that target SpAdK-mediated encapsulation to prevent pneumococcal infection.

## Electronic supplementary material

SUPPLEMENTARY MATERIALS AND RESULTS
