# Supplementary material for: Adenylate kinase potentiates the capsular polysaccharide by modulating Cps2D in Streptococcus pneumoniae D39
Source: Exp Mol Med. 2018 Sep 5;50(9):116. doi: 10.1038/s12276-018-0141-y (PMC6123713; doi:10.1038/s12276-018-0141-y)
Supplement: Supplementary file 1 — SUPPLEMENTARY MATERIALS AND RESULTS [file 12276_2018_141_MOESM1_ESM.docx]

**Adenylate kinase potentiates the capsular polysaccharide by modulating**

**Cps2D in *Streptococcus pneumoniae* D39**

Prachetash Ghosh^1#^, Truc Thanh Luong^1#^, Masaud Shah^2^, Trung Thanh Thach^3^, Sangdun Choi^2^, Sangho Lee^3^, Dong-Kwon Rhee^1^

^1^School of Pharmacy, Sungkyunkwan University, Suwon 16419, Korea

^2^ Department of Molecular Science and Technology, Ajou University, 16499, Korea

^3^Department of Biological Sciences, Sungkyunkwan University, Suwon 16419, Korea

^#^These authors contributed equally.

Corresponding author:

Dong-Kwon Rhee

Phone: (+82) 31 290 7707

Fax: (+82) 31 292 8800

E-mail: dkrhee@skku.edu

**Running title:** Modulation of the CPS by SpAdK

**Keywords:** SpAdK, capsular polysaccharide, Cps2D, ATP

**SUPPLEMENTARY MATERIALS AND METHODS**

## Ethical statement and raising antibodies

All of the animal experiments were performed according to protocol PH-530518-06 that was approved and monitored by the Animal Care and Use Committee of the Sungkyunkwan University (Suwon, Korea). This protocol strictly follows the animal care guidelines of the Korean Academy of Medical Sciences. The animals were maintained in a pathogen-free barrier facility (12 hr light/dark cycle, 22 ± 2°C, 50 ± 10% relative humidity) at the School of Pharmacy in Sungkyunkwan University. The male CD-1 (ICR) mice (4 weeks old, approximate 20 g each, and specific pathogen-free) were obtained from Orient Bio, Inc. (Seongnam, Gyeonggi, Korea) and acclimatized for a week. The animals were fed with water and sterile standard chow *ad libitum*.

For raising Cps2B, Cps2C, and Cps2D antibodies, 5-week-old male CD-1 mice were immunized intraperitoneally (*i.p*) with 10 µg purified protein combined with 100 µg aluminum adjuvant (Sigma-Aldrich, MO, USA) at 2-week intervals. One week after the third immunization, the mice were asphyxiated by CO_2_, and the sera were collected. The collected blood was kept at 25°C for 2 hr and then incubated at 4°C overnight. The sera were obtained after centrifugation at 13,000 × *g*, 4°C for 30 min. Later, the collected sera were filtered, and sodium azide (Sigma-Aldrich) was added to a final concentration of 0.2% to prevent fungal contamination. The antibody was stored at 4°C (for common use) or -70°C (for long-term storage).

## CLONING AND PURIFICATION OF PROTEINS

## *Cps2B and Cps2D (WT, 3Y3E, GK-AA, GK-AA/3Y3E)*

## The gene encoding Cps2B (GenBank ABJ54455.1) or Cps2D (GenBank ABJ55319.1) in *S. pneumoniae* type 2 D39 was amplified and inserted into a pET32b vector (Novagen) or His-2 parallel vector[^1^](#_ENREF_1) between the *Bam*HI and *Xho*I (Cps2B) or *Eco*RI (Cps2D) restriction enzyme sites to generate the pCPS01 or pCPS03 plasmid (Table 1). These recombinant plasmids were transformed to *E. coli* DH5α and subsequently transformed to *E. coli* BL21 (for pCPS01) or Rossetta2 (for pCPS03). The transformed *E. coli* strains were used to overexpress and purify His6-Cps2B protein and His6-Cps2D WT protein from inclusion bodies as described previously[^2^](#_ENREF_2)^,^ [^3^](#_ENREF_3). Briefly, *E. coli* cells (Novagen, Wisconsin, USA) were grown at 37°C until A_600_ = 0.5. Next, 0.1 mM IPTG was added to induce protein expression. The culture was further incubated at 16°C for 12 hr. The cells were harvested, resuspended in buffer A [150 mM NaCl, 50 mM Tris-HCl (pH 7.5), 0.1% Triton X-100, 1 mM DTT, and 1 mM EDTA], and lysed by sonication. The homogenate was centrifuged at 20,000 × *g* for 1 hr. Next, the pellet was washed twice with buffer A and resuspended in buffer B [250 mM NaCl, 50 mM Tris-HCl (pH 7.5), 0.1% Triton X-100, 0.5% sarkosyl, 1 mM DTT, 1 mM EDTA, and 1× protease inhibitor cocktail]. This was incubated at 4°C for 2 hr. The soluble fraction containing the protein was collected by centrifugation at 20,000 × *g* for 1 hr. The supernatant was applied to a Ni-NTA resin (GE Healthcare, Buckinghamshire, UK) and eluted in buffer C [500 mM NaCl, 50 mM Tris-HCl (pH 7.5), and 300 mM imidazole]. The protein was refolded by dialyzing the protein solution against buffer D [250 mM NaCl, 50 mM Tris-HCl (pH 7.5)] at 4°C for 12 hr. Next, the protein was further purified by Superdex 200 size exclusion chromatography (GE Healthcare) pre-equilibrated with buffer D. The refolded His6-Cps2D WT in buffer D was evaluated using dynamic light scattering with DynaPro 100 (Wyatt Technology, CA, USA) to characterize the monodispersity of the refolded proteins. The concentration of the pure protein was determined by measuring the A_280_.

The three tyrosine residues (Y215, Y218, and Y221) in the Y-cluster of Cps2D in the pCPS03 plasmid (Table 1) were mutated to glutamine (E) by using the Quikchange II site-directed mutagenesis kit (Agilent Technologies, California, USA) to generate the pCPS04 plasmid (named 3Y3E protein) and confirmed by DNA sequencing. The His6-Cps2D (3Y3E) protein was overexpressed and purified from the inclusion bodies following the same protocol used for the His6-Cps2D WT protein.

The two conserved residues (G48 and K49) in the ATP-binding domain of Cps2D in the pCPS03 and pCPS04 plasmids (Table 1) were mutated to alanine (A) by using the Quikchange II site-directed mutagenesis kit (Agilent Technologies, California, USA) to generate the pCPS05 and pCPS06 plasmids (named GA-KK and GA-KK/3Y3E proteins) and confirmed by DNA sequencing. The GA-KK and GA-KK/3Y3E proteins were overexpressed and purified from the inclusion bodies following the same protocol used for the His6-Cps2D WT protein.

***Cps2C***

## The gene encoding Cps2C (GenBank ABJ55335.1) in *S. pneumoniae* type 2 D39 was amplified and inserted into a pET32b vector (Novagen) or His-2 parallel vector (Novagen) between the *Bam*HI and *Xho*I restriction enzyme sites to generate the pCPS02 plasmid (Table 1). Next, the pCPS02 plasmid was transformed to *E. coli* DH5α and subsequently transformed to *E. coli* BL21 for overexpression and purification of His6-Cps2C as previously described[^4^](#_ENREF_4). Briefly, *E. coli* cells (Novagen, Wisconsin, USA) were grown at 37°C until A_600_ = 0.5. Next, 0.1 mM IPTG was added to induce protein expression. The culture was further incubated at 25°C for 12 hr. The cells were then harvested, resuspended in buffer E [150 mM NaCl, 50 mM Tris-HCl (pH 7.5), 0.1% Triton X-100], and lysed by sonication. The homogenate was centrifuged at 20,000× *g* for 1 hr, and the lysate was applied to a Ni-NTA resin (GE Healthcare, Buckinghamshire, UK) and eluted in buffer F [150 mM NaCl, 50 mM Tris-HCl (pH 7.5), and 250 mM imidazole]. The protein was dialyzed against buffer G [150 mM NaCl, 50 mM Tris-HCl (pH 7.5)] at 4°C for 12 hr. The concentration of the pure protein was determined by measuring the A_280_.

***SpAdK***

The gene encoding SpAdK (GenBank ABJ54173.1) in *S. pneumoniae* type 2 D39 was amplified and inserted into a pGST-parallel 2 vector (Novagen) to generate the pGST-ADK. SpAdK was purified from the *E. coli* strain expressing GST-SpAdK by glutathione affinity chromatography (GE Healthcare, Buckinghamshire, UK). The purified protein was dialyzed against buffer E [25 mM Tris-HCl (pH 7.5), 75 mM NaCl, 1 mM MgCl_2_, and 50 mM EDTA] overnight at 20°C. The protein was further purified in a Superdex-75 size exclusion column (GE HealthCare, Buckinghamshire, UK) equilibrated with buffer F [50 mM Tris-HCl (pH 7.5), 150 mM NaCl, and 1 mM MgCl_2_]. The fractions containing the pure GST-SpAdK were pooled and concentrated by 10-kDa cutoff centrifugal filters (Millipore). The monodispersity of the proteins was checked by dynamic light scattering on a DynaPro 100 (Wyatt Technology, CA, USA) in buffer F to confirm the molecular size of the pure GST-SpAdK. The protein concentration was determined by measuring the A_280_.

**Determination of bacterial growth doubling times**

*S. pneumoniae* was cultured in CAT media (for non-encapsulated strain) or THY media (for encapsulated strain) in the presence or absence of fucose (Sigma-Aldrich) (1.5 × 10^7^ CFU inoculate/5 mL media). Fucose was dissolved in CAT or THY media to a final concentration of 5% (w/v) as a stock, filtered through a 0.22-µm filter (Merck Millipore, MA, USA), and used immediately for experiment or stored at 4°C for future use. The A_550_ was measured every hour with a Novaspec III Visible Spectrophotometer (Amersham Biosciences, UK). The doubling time was calculated as the average time for a two-fold increase of the optical density at the logarithmic growth phase.

**qRT PCR**

In order to see the mRNA expression of the genes of *cps* operon in the D39 and TTL01 strains after independent exposure to the desired concentration of fucose, these strains were subjected to RNA isolation during the logarithmic growth phase. 1 µg of RNA was used as template for making cDNA in this study according to manufacturer’s protocol (Takara Clontech, CA, USA). The qRT-PCR reaction was carried out in a 20 µl final volume with the StepOnePlus Real-Time PCR System (Applied Biosystems, CA, USA) under the following reaction conditions comprising 100 ng of cDNA, 10 pmol of each primer (all the primers are listed in Table S1), and SYBR Green master mix (Elpis, Seoul, Korea). Cyclic conditions were 95 °C for 10 min followed by 40 cycles at 95°C for 15 s, 55°C for 30 s, and 72°C for 30 s. All the real-time PCR data were normalized to the mRNA level of 16srRNA gene as an internal control.

**Adherence and survival assays**

The adherence assay was performed as described previously with minor modifications[^5^](#_ENREF_5). Confluent monolayers of macrophages (RAW 264.7) or epithelial cells (A549 and HEp-2) were grown in 24-well plates (for adherence) or 6-well plates (for survival) in complete DMEM media. The media was replaced with DMEM media without antibiotics prior to infection with the pneumococci (MOI, 100) in the presence of fucose (0.1, 0.5, or 1.0%). To enumerate the total number of adherent and intracellular bacteria, the infected cells were lysed with 0.025% Triton X-100 in PBS, and the viable cell numbers were determined by plating on THY agar plates with appropriate fucose concentrations after dilution. For the survival assay, the RAW 264.7 macrophages were infected with the D39 or TTL01 strain, similar to the adherence assay. However, to eliminate the extracellular bacteria, the infected cells were incubated in complete medium containing 10 µg/mL penicillin and 200 µg/mL gentamicin for 1 hr and further incubated in complete media for 1 hr before plating. To enumerate intracellular pneumococci, supernatants were removed, and the infected cells were lysed with Triton X-100 as described above followed by serial dilution and plating on THY agar with the appropriate fucose concentration. All the samples were assayed in triplicate, and each assay was repeated at least twice.

**Fluorescence microscopy**

For visualization of the bacterial adherence, *S. pneumoniae* was grown exponentially in THY broth, labeled with FITC (Sigma-Aldrich) as described previously[^6^](#_ENREF_6), and infected onto confluent monolayers of the host cells (HEp-2 and RAW 264.7). After 2 hr of infection, the nuclei were stained with Hoechst (Sigma-Aldrich) and visualized by a confocal microscope (Carl Zeiss LSM 700 meta DuoScan, Jena, Germany).

**Phagocytic activity assay**

The previously described method for assessing the phagocytic activity[^7^](#_ENREF_7) was used with some modifications. The RAW 264.7 macrophages were infected with the pneumococci (MOI, 100) or zymosan (5 × 10^6^ particles/cell) in the absence or presence of fucose for the time-periods specified. The cells were then incubated with 600 µg/mL nitro blue tetrazolium (Sigma-Aldrich) for 1 hr. Subsequently, the supernatant was discarded, the pellet was dissolved in dimethyl sulfoxide (Katayama Chemical, Osaka, Japan) by light agitation, and the A_540_ was measured.

**SUPPLEMENTARY RESULTS**

**CPS synthesis requires SpAdK**

To study impact of SpAdK on pneumococcal growth, we determined growth rate using encapsulated and non-encapsulated strains. If CPS synthesis requires ATP via SpAdK, then growth rate of the encapsulated strain would be slower than the non-encapsulated one. When the doubling times of *adk* pneumococcal strains were compared (at 0.1% fucose concentration), the doubling time of encapsulated TTL01 (111 min) was 1.8-times slower than that of the non-encapsulated TTL04 (61 min; Supplementary Figure S1). This result suggests that low SpAdK levels at 0.1% fucose can generate sufficient ATP for the growth of the non-encapsulated strain but not for the encapsulated type. Therefore, the amount of ATP required for CPS synthesis may retard the growth of the *adk* mutant, encapsulated strain.

**SpAdK regulates the expression of type specific genes of *cps* operon**

It is already established that transcript levels of the *cps* locus are positively associated with CPS level, and *cps2A* gene has usually been used as a marker of the extent of transcription of pneumococcal *cps* locus[^8^](#_ENREF_8). As SpAdK plays a major role in the rise of pneumococcal CPS level, in order to see whether the *cps* locus expression is transcriptionally regulated by SpAdK, we examined the transcription of all the type specific genes along with *cps2A* gene in D39 WT and TTL01 strains using qRT-PCR (Supplementary Figure S2; results of only one representative from each type of genes are shown). The expression levels of these genes were minimum 14-fold higher in 1% fucose supplemented TTL01 strain as compared to that of D39 WT strain (data not shown). In addition, the expression of *cps2A* in 1% fucose supplemented TTL01 strain was increased about 142-fold in comparison to that of D39 WT strain (Supplementary Figure S2).

**Cps2D-oligomerization and autophosphorylation mechanism**

To understand the oligomerization capacity and autophosphorylation mechanism of Cps2D, the ADP- and ATP-bound states of Cps2D were docked and investigated. The top ten dimeric Cps2D complexes provided by each, MOE, ZDOCK and PatchDock, were vetted for the plausibility of its oligomerization. While considering the oligomeric states of CapB and Wzc as previously reported, only PatchDock provided similar but not identical conformers. Among the top ten refined solutions provided by FireDock, solution number seven was suggested to be the most plausible oligomeric form of Cps2D with lowest binding free energy (-2.12 kJ/mol) (Table S2). Regardless of the phosphorylated state of adenosine (ATP/ADP), PatchDock provided identical results for some particular conformers exhibiting lowest binding energy. Detailed interface analyses suggested that Cps2D interacts in a head-to-tail asymmetric manner as suggested in the previously reported BY-kinases. The tyrosine rich C-terminus motif of one monomer establishes interaction with the active site of the adjacent monomer. It was reported that EX2RX2R motif of α2 helix in BY-kinases stabilizes their oligomeric states[^9^](#_ENREF_9). However, we did not find this motif in Cps2D, instead we found that Tyr20 and Ser31 in the same helix participate in the oligomer stability through electrostatic interactions (Supplementary Figure S5A).

This model suggested that among the three tyrosine, Tyr215 and Tyr221 could possibly be phosphorylated (Supplementary Figure S5C). The hydroxyl group of Tyr215 points to the bound nucleotide in the active site suggesting its high probability to be initially phosphorylated.

Comparative analyses proposed that although Cps2D suggestively acquires a head to tail oligomerizing pattern; this orientation is somewhat different than that of previously reported BY-kinases. Upon superimposition with the *E. coli* Wzc tyrosine kinase, we observed that α2 of Cps2D faces the ATP binding pocket of the neighboring subunit. In addition to electrostatic contacts made by Ser31 and Tyr20 of α2, the oligomeric state seems to be partly stabilized by Val 203 and Leu205 in β6. Conversely, EX2RX2R motif of α2 helix in Wzc interacts with the glutamic acid in α7 and arginine in α2-β1 loop of the neighboring partner. This discrepancy can be explained by the fact that Cps2D lacks the charged “EX2RX2R” motif in its N-terminus. Sharing of considerable interface similarity with previously reported structures and successfully explaining the C-terminus autophosphorylation mechanism; we believe this could be the plausible model of Cps2D in ologomeric and monomeric form.

**Supplementary Figure S1**


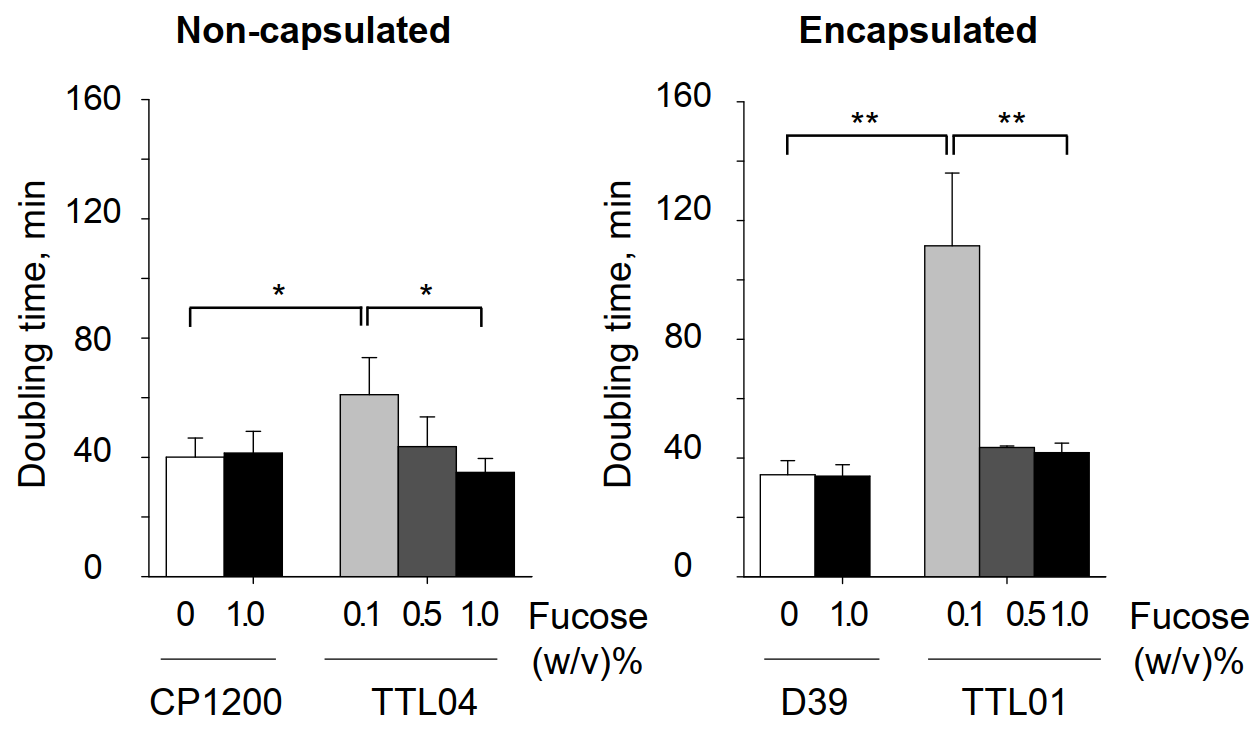


Supplementary Figure S1. Low expression of SpAdK caused longer doubling time in both encapsulated and non-encapsulated strains. *S. pneumoniae* growth was measured by optical density at A_550_. Optical density values as a function of time in the logarithmic growth phase were converted to doubling time. The error bars indicate standard deviations from three independent experiments. Significant differences were analyzed by One Way ANOVA (**P* ≤ 0.05 and ***P* ≤ 0.01).

**Supplementary Figure S2**


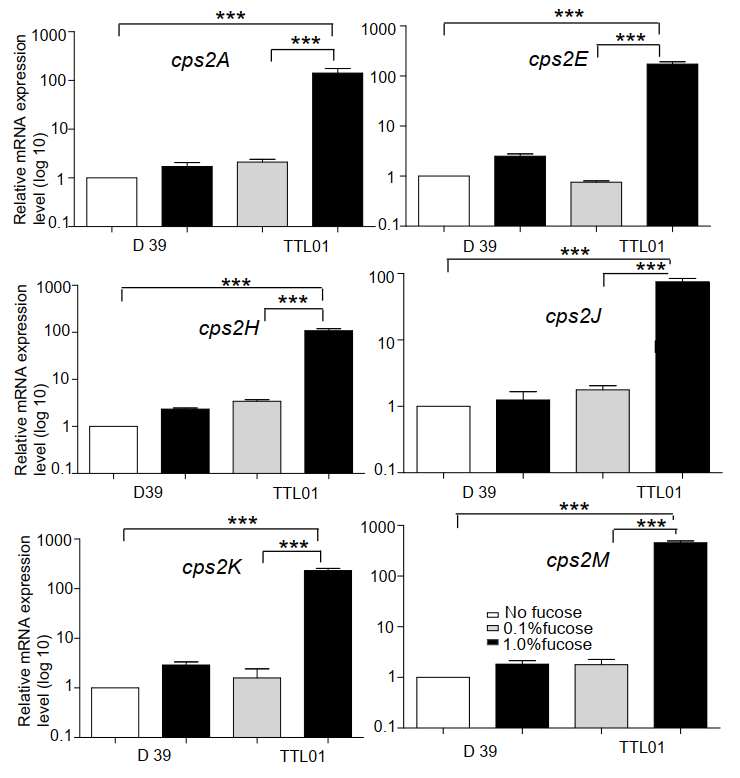


**Supplementary Figure S2. SpAdK induces capsule genes expression.** The expression profiles of *cps2A* (transcription activator) and one representative gene from each type-specific genes of *S. pneumoniae* mutant strain TTL01 and wild type D39 strain after fucose supplementation were analyzed by qRT-PCR. Data are expressed as mean ± standard error of mean (SEM) of 3 experiments in quadruplicates. **P* ≤ 0.05 (one-way ANOVA) as compared between groups

**Supplementary Figure S3**


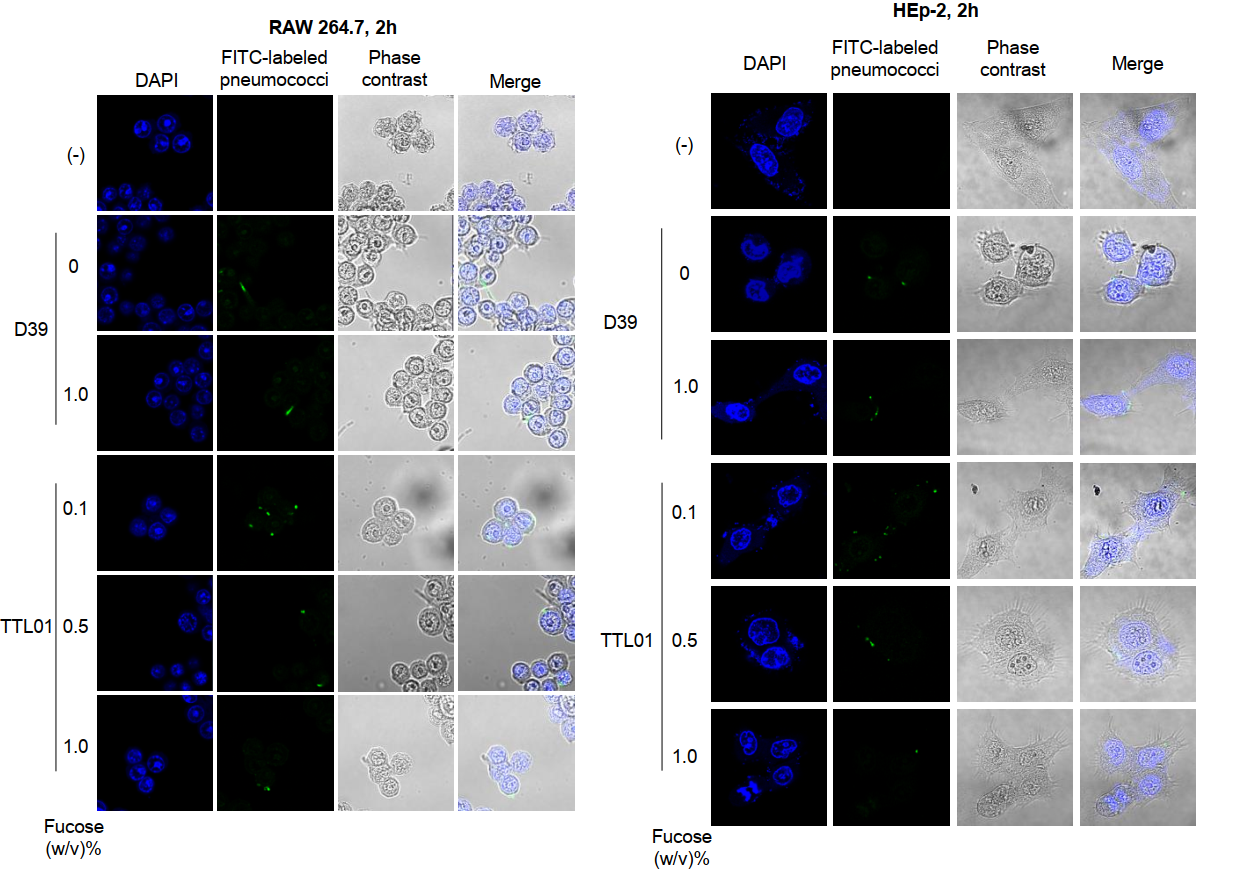


**Supplementary Figure S3. Impairment of *S. pneumonia*e adherence to the host cells by SpAdK.** Confluent monolayers of RAW 264.7 or HEp-2 cells were infected with the FITC-labeled pneumococcal strains at various concentrations of fucose for 2 hr. The adherent bacteria were visualized by confocal microscopy.

**Supplementary Figure S4**

**
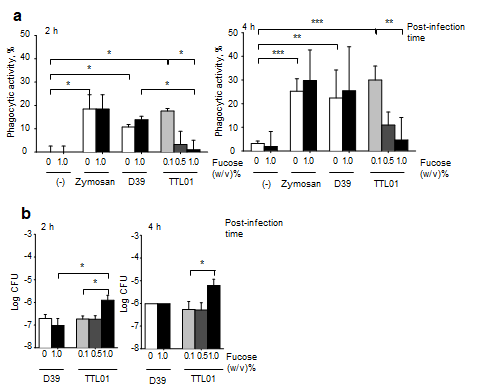
**

**Supplementary Figure S4. SpAdK enhances the resistance to phagocytosis**. (a) RAW 264.7 cells were infected with the pneumococcal strains (MOI, 100) at various concentrations of fucose or incubation times. The phagocytic activity was determined after infection of the RAW 264.7 cells with either the D39 or ∆*adk* (MOI, 100) strain fucose dose- and time-dependently. Zymosan was used as the bacterial control. (b) RAW 264.7 cells were infected with the pneumococcal strains (MOI, 100), at various concentrations of fucose or incubation times, and the extracellular pneumococci were eliminated by incubating with antibiotics; subsequently, the infected cells were incubated in the media for 1 hr before enumeration of the viable bacteria. The figure shows standard deviation from three independent experiments. Significant differences were analyzed by one-way ANOVA (**P* ≤ 0.05, ***P ≤* 0.01, and ****P ≤* 0.001).

**Supplementary Figure S5**

**
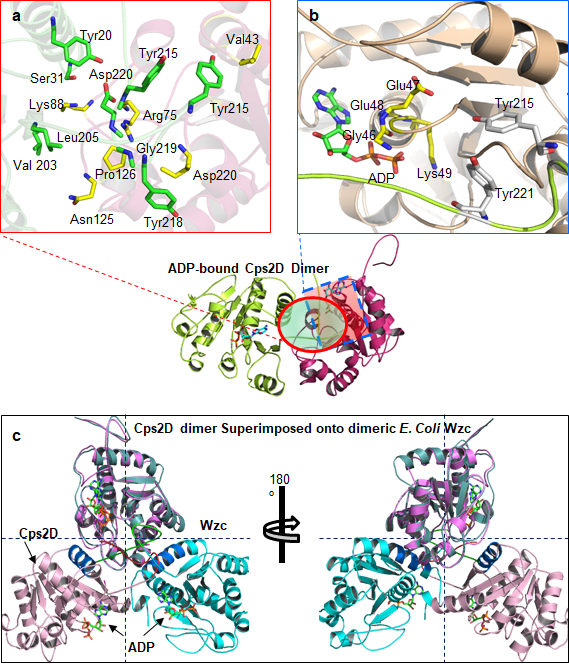
**

**Supplementary Figure S5. Cps2D oligomerization and auto-phosphorylation mechanism**. (a) The binding interface of Cps2D dimer (red box). (b) The C-terminus of Cps2D adjusts onto the ATP binding pocket that possibly regulate its autophosphorylation (blue box). (c) Comparative dimerization analysis of Cps2D with Wzc. The blue highlighted helix represents α2 in both proteins containing EX2RX2R motif in Wzc and absent in Cps2D.

**REFERENCES**

1. Sheffield P, Garrard S, Derewenda Z. Overcoming expression and purification problems of RhoGDI using a family of “parallel” expression vectors. *Protein Expr Purif* 1999; **15**(1)**:** 34-39.

2. Tao H, Liu W, Simmons BN, Harris HK, Cox TC, Massiah MA. Purifying natively folded proteins from inclusion bodies using sarkosyl, Triton X-100, and CHAPS. *Biotechniques* 2010; **48**(1)**:** 61-64.

3. Drew D, Lerch M, Kunji E, Slotboom D-J, de Gier J-W. Optimization of membrane protein overexpression and purification using GFP fusions. *Nature Methods* 2006; **3**(4)**:** 303-313.

4. Luong TT, Kim E-H, Bak JP, Nguyen CT, Choi S, Briles DE*, et al.* Ethanol-induced alcohol dehydrogenase E (AdhE) potentiates pneumolysin in *Streptococcus pneumoniae*. *Infect Immun* 2015; **83**(1)**:** 108-119.

5. Kwon H-Y, Ogunniyi AD, Choi M-H, Pyo S-N, Rhee D-K, Paton JC. The ClpP protease of Streptococcus pneumoniae modulates virulence gene expression and protects against fatal pneumococcal challenge. *Infect Immun* 2004; **72**(10)**:** 5646-5653.

6. Nguyen CT, Le N-T, Tran TD-H, Kim E-H, Park S-S, Luong TT*, et al.* *Streptococcus pneumoniae* ClpL modulates adherence to A549 human lung cells through Rap1/Rac1 activation. *Infect Immun* 2014; **82**(9)**:** 3802-3810.

7. Okimura T, Ogawa M, Yamauchi T, Sasaki Y. Stress and Immune Responses IV. Adrenal Involvement in the Alteration of Antibody Responses in Restraint-Stressed Mice. *Jpn J Pharmacol* 1986; **41**(2)**:** 237-245.

8. Wu K, Xu H, Zheng Y, Wang L, Zhang X, Yin Y. CpsR, a GntR family regulator, transcriptionally regulates capsular polysaccharide biosynthesis and governs bacterial virulence in *Streptococcus pneumoniae*. *Sci Rep* 2016; **6:** 29255.

9. Bechet E, Gruszczyk J, Terreux R, Gueguen-Chaignon V, Vigouroux A, Obadia B*, et al.* Identification of structural and molecular determinants of the tyrosine-kinase Wzc and implications in capsular polysaccharide export. *Mol Microbiol* 2010; **77**(5)**:** 1315-1325.

**Table S1. Primers used in this study**

| **Primer name** | **Sequence (5’-3’**) |
| --- | --- |
| cps2A (F) | CGTCAACCGAAGCACTG |
| cps2A (R) | GATCCATCCGACCTGTCC |
| cps2E (F) | ATGGGGGCTCTGCTATTTTT |
| cps2E (R) | TCCACCCTGCATGGTATTTT |
| cps2H (F) | ATGGACTGGCTGATGGTTCT |
| cps2H (R) | TTGACACCACCAAATCCTGA |
| cps2J (F) | TTTTGTGGTGTAGCGGTGAA |
| cps2J (R) | ATTCCACCAGCAAATCCTGA |
| cps2K (F) | TGAACGCTTGGTAAGTGCTG |
| cps2K (R) | ACCGCCTCAGCTTCAGTAAA |
| cps2M (F) | CGTGGCTGGTTTAAGGAAAA |
| cps2M (R) | TCAACCCAAGAACCCAGAAC |

**Table S2. The FireDock output table for the PatchDock solutions. Solutions are ranked by the global energy value.**

| **Rank** | **Solution number** | **Global energy** | **Attractive VdW** | **Repulsive VdW** | **ACE** | **HB** |
| --- | --- | --- | --- | --- | --- | --- |
| 1 | 7 | -2.12 | -24.97 | 12.03 | 1.97 | -2.13 |
| 2 | 5 | 5.98 | -10.43 | 2.26 | 4.11 | 0 |
| 3 | 2 | 7.1 | -25.56 | 16.43 | 6.12 | -3.11 |
| 4 | 10 | 7.41 | -36.8 | 10.69 | 13.74 | -1.92 |
| 5 | 4 | 7.47 | -0.58 | 0 | 1.3 | 0 |
| 6 | 1 | 11.39 | -2.29 | 0 | -0.05 | 0 |
| 7 | 6 | 34.38 | -51.25 | 45.89 | 15.11 | -1.85 |
| 8 | 8 | 35.42 | -27.09 | 20.24 | 15.27 | -1.11 |
| 9 | 3 | 42.54 | -25.29 | 17.92 | 17.58 | -2.97 |
| 10 | 9 | 426.37 | -30.48 | 536.73 | 13.97 | -1.14 |

**Rank** - according to the global energy. **Solution Number** - according to the order of the solutions provided by PatchDock. **Global Energy** - The binding energy of the solution. **Attractive and Repulsive VdW** – The contribution of the van der Waals forces to the global binding energy. **ACE** - The contribution of the atomic contact energy (ACE) to the global binding energy. **HB** - The contribution of the hydrogen bonds to the global binding energy.
